# Medico-legal aspects of fatal ricin poisoning: a systematic review of clinical manifestations, toxicological findings and autopsy results

**DOI:** 10.1007/s00414-026-03949-0

**Published:** 2026-08-06

**Authors:** Isabella Aquila, Matteo Antonio Sacco

**Affiliations:** Institute of Legal Medicine, Department of Medical and Surgical Sciences, Magna Graecia University, Catanzaro, 88100 Italy

**Keywords:** Ricin, Ricinine, Fatal poisoning, Forensic toxicology, Autopsy, Biomarker

## Abstract

Ricin, a potent protein toxin derived from *Ricinus communis*, is considered one of the most significant threats in cases of bioterrorism, homicide, suicide, and accidental acute poisoning. However, its clinical and pathological features in fatal cases have not been systematically summarized. This systematic review aimed to summarize the current evidence regarding the clinical manifestations, toxicological biomarkers, autopsy findings, and medico-legal aspects of fatal ricin poisoning. A comprehensive literature search was conducted in accordance with PRISMA guidelines. Original studies, case reports, and case series reporting detailed clinical, toxicological, or autopsy data following fatal or near-fatal human exposure to ricin were included in the review. The available evidence suggests a relatively consistent clinical course, characterized by rapid progression from mild, nonspecific gastrointestinal symptoms to hypovolemic shock, multi-organ failure, and refractory cardiovascular collapse. Ricinine was consistently detected in blood, urine, and postmortem tissues, supporting its role as a reliable biomarker of ricin exposure, although current evidence is insufficient to establish definitive correlations with poisoning severity or toxicokinetics. Reported autopsy findings commonly included hemolysis, hemorrhagic necrosis of the gastrointestinal tract, hepatic injury, renal tubular necrosis, and evidence supporting the presence of a capillary leak syndrome. Several specific medico-legal challenges emerged from the reviewed literature, including the need for the development of novel analytical methods with improved detection limits and quantification capabilities for ricinine, the interpretation of postmortem redistribution phenomena, the distinction between lethal and non-lethal exposures, and the differential diagnosis with other septic shock-like syndromes. Overall, the available evidence suggests a relatively consistent clinicopathological pattern associated with fatal ricin poisoning that can be corroborated through ricinine detection, while emphasizing the importance of early toxicological investigations, particularly when no history of exposure is available. These findings provide a concise and integrated body of evidence to support clinical management, inform forensic investigations, and enhance public health preparedness, while highlighting the need for standardized postmortem sampling protocols and further research into the toxicodynamics of ricin in humans.

**Graphical abstract:**

**Figure:**
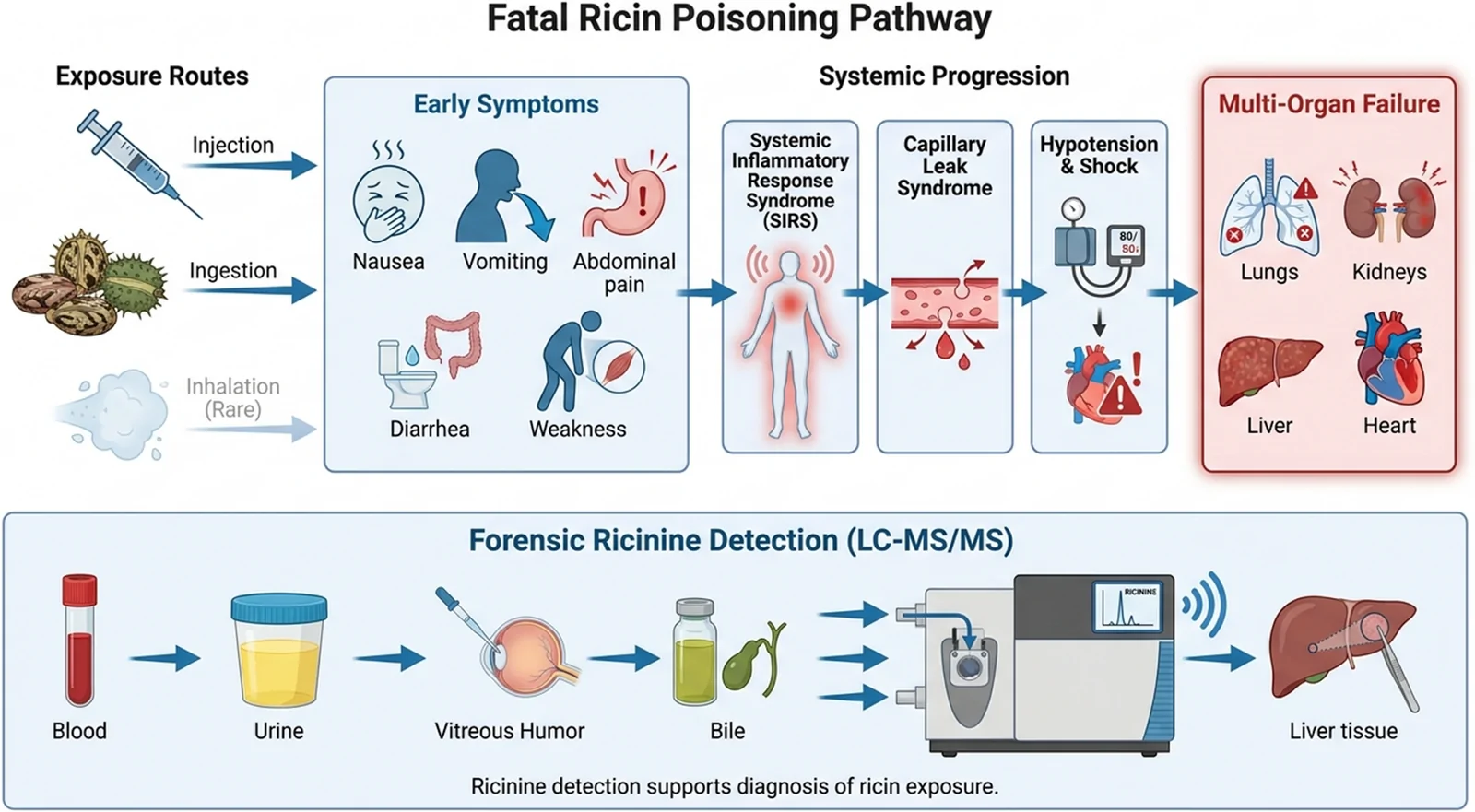

**Supplementary Information:**

The online version contains supplementary material available at https://doi.org/10.1007/s00414-026-03949-0.

## Introduction

 Ricin is a potent type 2 ribosome-inactivating protein (RIP) derived from the seeds of the castor oil plant (*Ricinus communis*) and has long been recognized as a highly lethal poison [1]. Its high toxicity, low production cost, and environmental stability have made it a persistent concern for public health authorities, law enforcement agencies, and national security organizations worldwide [2]. Ricin exerts its toxic effects by removing a single adenine residue from 28 S ribosomal RNA, resulting in irreversible inhibition of protein synthesis and the activation of secondary cellular stress pathways that ultimately lead to cell death [3]. Although the molecular mechanisms underlying ricin toxicity have been extensively investigated in vitro and in animal models, their translation to the clinical and pathological manifestations observed in human fatal poisoning remains incomplete and fragmented [4].

Ricin poisoning has long been associated with targeted assassinations, most notably the widely reported 1978 death of Bulgarian dissident Georgi Markov, which has generally been attributed to ricin delivered through a modified umbrella, although definitive toxicological confirmation has never been established. More recently, several incidents involving ricin-contaminated letters sent to government officials have highlighted the continued threat posed by this toxin [5]. These events illustrate the diverse circumstances under which ricin may cause fatal poisoning, including deliberate homicidal use, accidental laboratory exposures, and, less frequently, intentional self-administration [6]. However, the medical and forensic literature on human ricin poisoning remains largely limited to individual case reports and small case series, with no comprehensive synthesis of the clinical course, diagnostic findings, and autopsy features associated with fatal outcomes [7]. The absence of a centralized evidence base represents a major challenge for clinicians faced with unexplained toxic exposures, for forensic pathologists tasked with determining the cause and manner of death, and for public health authorities responsible for preparedness against potential bioterrorism events.

Several important research gaps continue to limit our understanding of fatal ricin poisoning. First, the clinical progression from exposure to death has never been systematically characterized according to the route of intoxication, despite the fact that ingestion, inhalation, and injection each involve distinct toxicokinetic and toxicodynamic profiles [8]. Second, ricinine, a low-molecular-weight alkaloid naturally associated with castor beans and commonly used as a biomarker of ricin exposure, has been detected in multiple biological matrices; however, no comprehensive human study has definitively established the relationship between ricinine concentrations, poisoning severity, and time since exposure [9]. Third, autopsy findings in fatal ricin poisoning, including patterns of organ injury and characteristic histopathological alterations, have been described only in a limited number of reports, resulting in the absence of standardized forensic criteria for diagnosis in cases of unexplained death [8]. Fourth, several medico-legal aspects of ricin poisoning, including postmortem redistribution phenomena [9], the analytical methodologies required to confirm toxin exposure, and the potential for misdiagnosis as septic shock or other toxidromic conditions, remain insufficiently explored in the literature [10].

Therefore, the aim of this systematic review is to address these major gaps in knowledge by systematically evaluating and synthesizing the available evidence regarding the clinical manifestations of fatal ricin poisoning, toxicological biomarkers detected following lethal exposure, autopsy findings documenting tissue damage caused by ricin toxicity, and the medico-legal implications associated with fatal ricin exposure. By collecting and analyzing all available human data in a systematic manner, we aim to integrate current knowledge into a coherent model of lethal ricin poisoning, spanning the continuum from initial symptom onset to the final pathological outcome. This review is intended to serve as a valuable resource for clinicians involved in the early recognition and management of ricin toxicity, for forensic pathologists responsible for establishing the diagnosis at autopsy, and for public health and security professionals engaged in preparedness planning and response to potential ricin-related incidents.

## Materials and methods

### Review protocol

This systematic review was conducted in accordance with the Preferred Reporting Items for Systematic Reviews and Meta-Analyses (PRISMA) guidelines [11]. A comprehensive literature search was performed up to 1 June 2026 across three major electronic databases: PubMed, Scopus, and Web of Science.

For each database, the search strategy was adapted according to the specific indexing system and search functionality. Multiple combinations of controlled vocabulary (where available) and free-text keywords related to ricin exposure, poisoning, clinical manifestations, toxicology, pathology, autopsy, and forensic medicine were used to maximize the sensitivity and comprehensiveness of the search. In PubMed, Medical Subject Headings (MeSH) were incorporated whenever appropriate and combined with free-text terms. Equivalent search strategies were adapted for Scopus (TITLE-ABS-KEY) and Web of Science (TOPIC). The representative search strategies are provided in Supplementary Table S1.

In addition, the reference lists of all studies meeting the eligibility criteria were manually screened to identify any potentially relevant publications that had not been retrieved through the electronic database search. No date restrictions were applied in order to capture both historical and contemporary reports of human ricin poisoning. Only studies published in English were considered eligible for inclusion.

The core search string was as follows:

- ((Ricin OR “Ricinus communis” OR “castor bean”) AND (poisoning OR intoxication OR toxicity OR fatal OR death) AND (clinical manifestations OR symptoms OR toxicokinetics OR biomarkers) AND (autopsy OR pathology OR postmortem OR histopathology) AND (forensic OR medico-legal OR legal implications OR criminal))

### Classification of exposure routes

Given the substantial heterogeneity of reported cases, studies were categorized according to the route of exposure. This approach was considered essential because the toxicokinetic behavior, clinical evolution, and pathological consequences of ricin poisoning vary considerably depending on how the toxin enters the body.

Cases involving injection were evaluated separately because direct inoculation into tissues or the bloodstream results in rapid systemic distribution and is typically associated with severe toxicity and a high fatality rate. Oral exposure cases, most frequently resulting from the ingestion of castor beans or ricin-containing preparations, were analyzed as a distinct category because they are characterized by variable gastrointestinal absorption, delayed symptom onset, and a different pattern of systemic involvement. Less common routes of exposure, including inhalational and dermal contact, were grouped together owing to the limited number of documented cases. Despite their rarity, these routes remain highly relevant from a public health and bioterrorism preparedness perspective.

This classification framework enabled a structured comparison of clinical manifestations, toxicological findings, and pathological alterations across different exposure modalities while also facilitating the identification of common features shared among fatal cases.

### Inclusion and exclusion criteria

Strict eligibility criteria were established to ensure the methodological consistency and clinical relevance of the studies included in this review. Eligible publications comprised original research articles, case reports, case series, observational studies, experimental toxicology investigations, and systematic reviews or meta-analyses. Studies were considered suitable for inclusion when they described fatal or near-fatal human ricin poisoning and provided detailed information regarding clinical manifestations, toxicological biomarkers, autopsy findings, histopathological alterations, or medico-legal implications. Human subjects of any age, sex, or ethnic background were eligible, provided that the diagnosis of fatal ricin poisoning had been analytically confirmed or was strongly supported by clinical, toxicological, and circumstantial evidence.

Included studies were also required to report at least one relevant outcome measure, such as the temporal progression of clinical symptoms, qualitative or quantitative toxicological findings in biological fluids or postmortem tissues, histopathological observations, or information relevant to the determination of the cause and manner of death. Only peer-reviewed articles published in scientific journals, available in full text, and written in English were considered eligible for data extraction.

Studies were excluded when they focused exclusively on animal models or in vitro experiments without direct relevance to human fatal poisoning. Reports describing non-fatal exposures without life-threatening manifestations were not considered eligible because the objective of the review was to characterize fatal and near-fatal intoxications. Narrative reviews, editorials, commentaries, and letters to the editor lacking original case data were excluded. In instances of duplicate publication, only the most comprehensive and detailed report was retained for analysis. Articles providing insufficient clinical, toxicological, pathological, or autopsy information to support a diagnosis of ricin poisoning were also excluded. Likewise, conference abstracts, scientific communications without a corresponding peer-reviewed full-text publication, and manuscripts available exclusively through preprint repositories were not considered eligible for inclusion.

### Study selection process

Study selection was conducted according to PRISMA recommendations to ensure a transparent and reproducible review process. Two independent reviewers screened all retrieved records at the title and abstract level according to the predefined eligibility criteria. Any discrepancies arising during the screening process were resolved through discussion and consensus.

All references were imported into reference management software (Zotero) to facilitate record organization and duplicate removal. The initial database search identified 240 potentially relevant records. Following the removal of 84 duplicate entries, 156 unique records remained for title and abstract screening. Of these, 92 records were excluded because they did not meet the eligibility criteria, most commonly due to their focus on non-human studies, non-fatal intoxications, or topics unrelated to ricin poisoning.

The full texts of the remaining potentially eligible studies were subsequently assessed. Although 64 articles were initially considered for full-text review, only 51 could be retrieved despite repeated attempts through institutional access, interlibrary loan services, and author contact. The remaining 13 articles were therefore excluded because the full text was unavailable.

Full-text evaluation led to the exclusion of an additional 41 studies. The most frequent reasons for exclusion included the absence of original case data, insufficient clinical, toxicological, or autopsy information, duplicate publication of previously reported cases, conference abstracts lacking a corresponding peer-reviewed full-text article, and articles published in languages that could not be reliably translated for data extraction purposes.

**Fig. 1:**
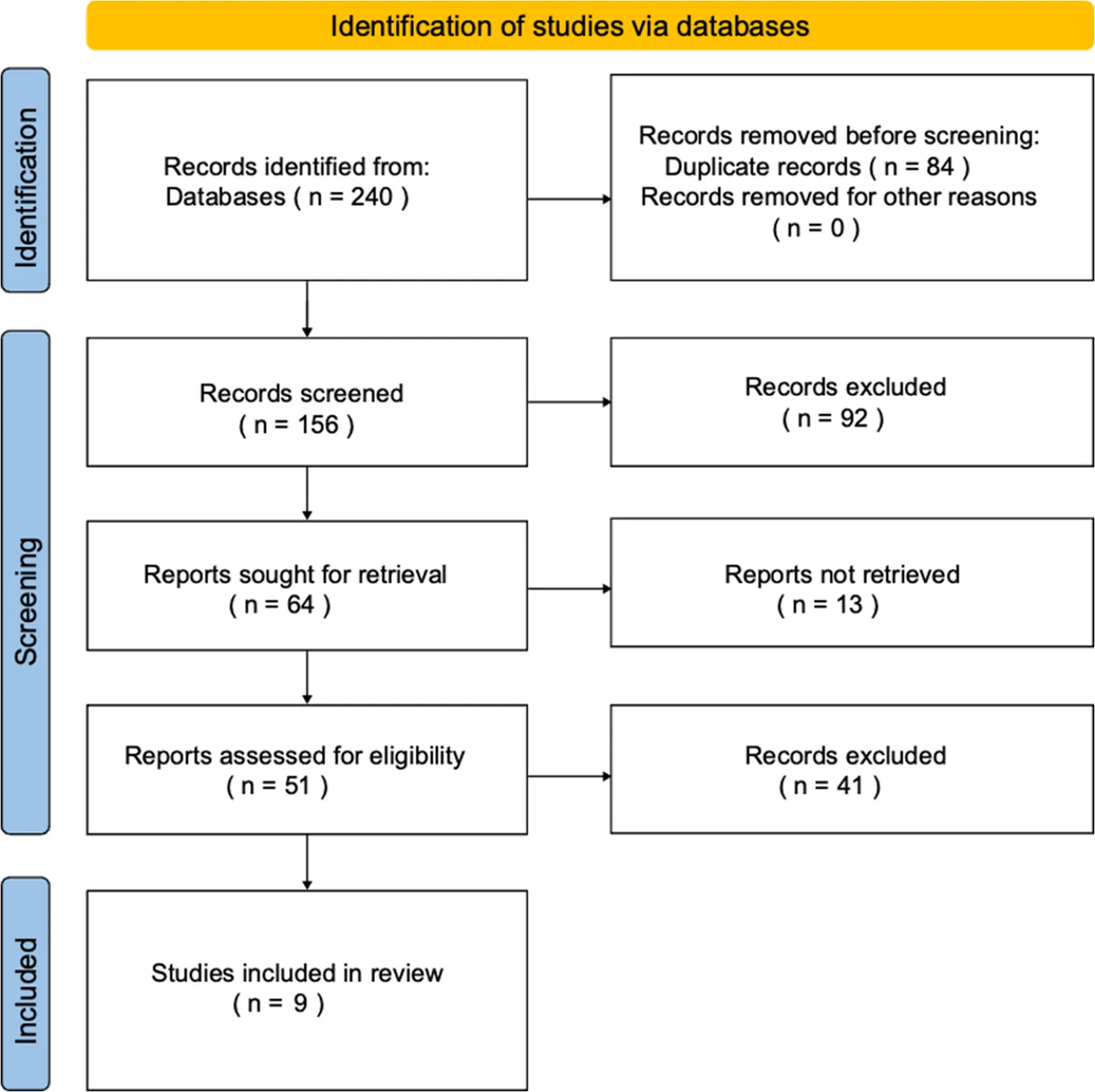
PRISMA flow chart of the study selection process

Ultimately, 9 studies fulfilled all predefined eligibility criteria and were included in the final qualitative synthesis. The study selection process is summarized in the PRISMA flow diagram presented in Fig. 1.

### Quality assessment and limitations

The methodological quality of the included studies was assessed using a modified version of the Joanna Briggs Institute (JBI) Critical Appraisal Checklist for Case Reports and Case Series. The appraisal focused on the clarity of the clinical history, the adequacy of laboratory investigations, including toxicological confirmation of ricin exposure, the completeness of autopsy and histopathological descriptions, and the discussion of relevant medico-legal aspects. Based on these criteria, each study was categorized as having a low, moderate, or high risk of bias. The results of the methodological quality assessment are summarized in Supplementary Table S2.

Studies were not excluded solely on the basis of methodological quality. Instead, the quality assessment was used to support the interpretation of findings and to identify potential sources of bias and uncertainty within the available evidence.

Several limitations should be acknowledged. The restriction to English-language publications may have introduced language bias, potentially excluding relevant cases reported in other languages. Second, publication bias is likely to have affected the available literature, as unusual or particularly severe cases are more likely to be reported than cases with negative, inconclusive, or less distinctive findings. Also, the limited number of eligible studies precluded quantitative meta-analysis and restricted the generalizability of the findings. Finally, considerable variability in reporting quality and completeness among studies limited the availability of uniform data across all variables of interest.

## Results

The temporal distribution of publications demonstrates a persistent, although relatively limited, scientific interest in fatal ricin poisoning. Most of the available evidence originated from studies published before 2019, with five of the nine included studies appearing during this period. The remaining studies were published between 2019 and 2026. Although the overall volume of research remains small, the continued publication of new studies suggests an ongoing interest in ricin poisoning, driven in part by advances in analytical toxicology, improvements in biomarker detection, and persistent concerns regarding bioterrorism and public safety.

A notable finding emerging from this review is the absence of large-scale epidemiological investigations or multicenter studies specifically focused on fatal ricin poisoning. The predominance of isolated case reports and small case series reflects both the rarity of documented cases and the practical difficulties associated with studying a relatively uncommon and often clandestine form of poisoning. Nevertheless, the continued publication of recent studies indicates that research efforts remain active, particularly in areas related to novel analytical methodologies, biomarker identification, postmortem toxicology, and medico-legal investigation.

Despite this sustained interest, the identification of only nine eligible studies over several decades highlights the limited nature of the available evidence. This observation underscores the need for collaborative multicenter initiatives capable of collecting standardized clinical, toxicological, and pathological data from future cases, thereby strengthening the evidence base available for both clinical management and forensic practice.

### Overview of included studies

The characteristics of the included studies are summarized in Table 1. Data extraction focused on study design, population characteristics, route of exposure, toxicological biomarkers, analytical methods used for toxin detection, and clinical outcomes, including survival or death where applicable.

**Table 1:** Characteristics of Included Studies

Study	Study design	Population	Exposure route	Biomarkers detected	Toxicological analytical method	Clinical outcome
Aviram et al. [12]	Method development and validation with application to a forensic case	Single individual (suicide)	Self-injection of castor bean extract	Ricinine, L-abrine	LC–MS/MS using dried urine spot (DUS) and dried blood spot (DBS)	Death
Bucaretchi et al. [13]	Case report	21-year-old man	Intramuscular and subcutaneous injection	Ricin A-chain, ricinine, IL-6, IL-10, TNF-α, troponin, creatine kinase, hypoalbuminemia, proteinuria, thrombocytopenia, leukocytosis	ELISA (ricin A-chain); LC–MS (ricinine)	Near-fatal survival
Coopman et al. [14]	Case report	49-year-old man	Intravenous and subcutaneous injection	Ricinine (blood, urine, vitreous humour)	GC–MS, HPLC-PDA, LC–MS, LC–MS/MS	Death
Passeron et al. [15]	Case report	53-year-old man with depression	Subcutaneous injection of masticated castor beans	Not reported	Not reported	Near-fatal survival
Rauniyar et al. [16]	Case report	Pediatric patient	Castor seed ingestion	Not reported	Not reported	Survival
Røen et al. [17]	Case report	Adult patient	Mixed exposure (oral ingestion and injection)	Ricinine	Isotope-dilution LC–MS/MS following solid-phase extraction	Death
Staňková et al. [18]	Case study	Adult patient	Intravenous injection	Ricinine	GC–MS	Death
Steinacher et al. [19]	Case report	Previously healthy adult	Castor bean ingestion	Not reported	Not reported	Near-fatal survival
Verougstraete et al. [20]	Case report with analytical method validation	26-year-old man	Injection of castor bean extract	Ricinine (serum, blood, urine, bile, liver tissue)	LC–MS/MS after liquid-liquid extraction	Death

*LC–MS/MS* liquid chromatography–tandem mass spectrometry, *LC–MS* liquid chromatography–mass spectrometry, *GC–MS* gas chromatography–mass spectrometry, *HPLC* high-performance liquid chromatography, *ELISA* enzyme-linked immunosorbent assay

Considerable heterogeneity was observed across the included studies. Differences were identified in study design, exposure circumstances, route of intoxication, toxicological investigations, and reported outcomes. Such variability should be taken into account when interpreting the findings of this review, as it represents an important source of heterogeneity within the evidence base.

Despite these differences, the included studies collectively provide valuable information regarding the clinical presentation, toxicological confirmation, pathological findings, and medico-legal implications of fatal ricin poisoning.

### Injection exposure: Rapid clinical progression and ricinine as a key biomarker

Cases involving parenteral administration provide a unique opportunity to examine the most rapid and severe manifestations of ricin poisoning. Unlike oral exposure, injection bypasses gastrointestinal barriers and delivers the toxin directly into tissues or the systemic circulation, resulting in greater bioavailability and accelerated toxic effects. This route is of particular clinical and forensic importance because it has been associated with homicidal attacks, suicidal attempts, and deliberate bioterrorism-related incidents. The studies included in this review [12–15, 17, 18, 20] consistently describe a characteristic syndrome characterized by rapid clinical deterioration, reproducible toxicological findings, and distinctive pathological features.

Overall, injection-related poisoning showed a relatively homogeneous clinical presentation across the included studies. The most frequently reported manifestations included local inflammatory reactions at the injection site, early gastrointestinal symptoms, rapidly progressive hypotension, refractory shock, systemic inflammatory response syndrome, and multiple organ failure. Despite differences in the administered dose and exposure circumstances, fatal cases generally followed a similar clinical trajectory characterized by rapid deterioration within the first 24–48 h after exposure.

Across the included studies, ricinine was the biomarker most consistently investigated and was successfully detected in multiple biological matrices, including blood, serum, urine, vitreous humour, bile, and liver tissue. Other biomarkers, including ricin A-chain and inflammatory markers (e.g., IL-6, IL-10, TNF-α), were reported less frequently and mainly reflected systemic toxicity rather than providing exposure-specific confirmation. Overall, toxicological findings were remarkably consistent across studies, supporting the value of ricinine as the principal biomarker for confirming ricin exposure in both clinical and forensic settings.

Despite variations in the administered dose and the timing of medical intervention, the clinical course appeared broadly consistent across the reported cases. Initial symptoms generally developed within a few hours of exposure and typically included localized pain, swelling, and erythema at the injection site, followed by nonspecific systemic manifestations such as nausea, vomiting, abdominal cramps, malaise, and generalized weakness [13, 14, 20]. This prodromal phase was usually brief, with severe systemic manifestations emerging approximately two to six hours after exposure (Table 2). Verougstraete et al. [20] described severe abdominal cramps and nausea developed shortly after injection, whereas the patient described by Coopman et al. [14] experienced nausea, vomiting, diarrhea, dyspnea, vertigo, and myalgia within the first 24 h. Compared with oral poisoning, in which significant gastrointestinal symptoms may be delayed for 12–24 h after exposure, the accelerated onset observed following injection reflects the more rapid systemic distribution of ricin when directly introduced into highly vascularized tissues or the bloodstream.

**Table 2:** Summary of Clinical Progression in Injection Exposure Cases

Study	Time post-exposure to first medical contact	Initial symptoms	Progression to organ failure	Time to death or discharge	Ricinine detection and levels
[12]	Not specified (suicide)	Not specified	Not specified	Death	Ricinine detected in urine 72 h post-exposure (quantitative levels not provided)
[13]	1 h	Mild local pain, slight local edema and erythema	Refractory shock ~ 24 h post-exposure, systemic inflammatory response syndrome, multiple organ dysfunction, capillary leak syndrome, rhabdomyolysis, necrotizing fasciitis, compartment syndrome	Discharged on day 71	Ricin A-chain detected in serum up to day 3; ricinine detected in serum and urine up to day 5; ricin A-chain concentration in extract was 246 µg/mL
[14]	24 h	Nausea, vomiting, diarrhoea, dyspnoea, vertigo, muscular pain	Multi-organ failure	Death 9 h after admission (33 h post-exposure)	Ricinine identified in blood, urine, and vitreous humour using LC-MS/MS
[15]	Not specified	Cellulitis of the thigh	Not specified	Not specified	Not reported
[17]	4 h	Not specified	Multi-organ failure	Death 38 h post-exposure	Ricinine in serum declined from 33 ng/mL to 23 ng/mL between 10 and 29 h; urine levels 20–58 ng/mL (creatinine-corrected 21–30 µg/g)
[18]	Not specified	Not specified	Not specified	Death	Ricinine detected in urine and extract; approximately uniform postmortem distribution reported
[20]	5.5 h	Severe abdominal cramps, nausea	Fulminant diarrhea, hypovolemic shock, cardiorespiratory collapse, multiple organ failure	Death approximately 10 h after injection	Ricinine in serum 16.5 ng/mL and blood 12.9 ng/mL at 6 h; decreased to 12.4 and 10.6 ng/mL at 10 h; urine 81.1 ng/mL at 7 h; also detected in bile and liver tissue

Following the initial symptomatic phase, fatal cases included in this review progressed to refractory shock and multiple organ failure within a relatively short timeframe, ranging from approximately 10 h [20] to 33 h after exposure [14], although some reports described a more prolonged course. The survivor described by Bucaretchi et al. [13] exhibited a comparable clinical pattern but eventually recovered after intensive supportive treatment. Shock was typically characterized by profound hypotension that remained refractory to aggressive fluid resuscitation and high-dose vasopressor therapy, suggesting a complex pathophysiological process involving both severe capillary leakage and direct myocardial dysfunction [13, 20]. In the case reported, by Verougstraete et al. [20], the patient rapidly developed profuse diarrhea leading to hypovolemic shock and cardiorespiratory collapse within approximately 10 h. Similarly, the patient described by Coopman et al. [14] died from multiple organ failure only nine hours after hospital admission. Such rapid progression to circulatory collapse appears to be a recurrent feature of fatal injection-related ricin poisoning and distinguishes it from the generally more protracted clinical course observed following ingestion.

Another recurrent finding was the development of a systemic inflammatory response syndrome (SIRS)-like condition closely resembling severe septic shock. The surviving patient reported by Bucaretchi et al. [13] demonstrated markedly elevated circulating concentrations of pro-inflammatory cytokines, including interleukin-6 (IL-6), interleukin-10 (IL-10), and tumor necrosis factor-alpha (TNF-α), accompanied by leukocytosis and thrombocytopenia. These findings support the hypothesis that ricin is capable of triggering a profound inflammatory response independent of any infectious process. From a clinical and medico-legal perspective, this presentation represents a significant diagnostic challenge because the syndrome may be misinterpreted as bacterial sepsis, particularly when no history of ricin exposure is available.

### Ingestion exposure: Delayed onset, variable severity, and the diagnostic role of ricinine

Accidental ricin poisoning most commonly occurs following the ingestion of castor beans, particularly among children and individuals attempting self-harm. Unlike injection exposure, which delivers the toxin directly into tissues or the bloodstream, oral exposure subjects ricin to the hostile environment of the gastrointestinal tract. For toxicity to occur, the protective outer coating of the castor bean must be disrupted through chewing or mechanical fragmentation, allowing the toxin to be released. Furthermore, ricin is a large water-soluble protein that may undergo partial degradation by gastric acid and digestive enzymes, resulting in highly variable and often incomplete absorption. Consequently, oral poisoning is generally characterized by a delayed onset of symptoms, a more heterogeneous clinical presentation, and a lower fatality rate compared with injection-related exposures.

Exposure circumstances also differed substantially, including suicidal, accidental and homicidal poisonings, potentially contributing to variability in clinical presentation.

Compared with injection exposure, ingestion-related poisoning showed greater variability in clinical severity and outcome across the included studies. Gastrointestinal manifestations represented the predominant clinical presentation, whereas severe cardiovascular complications were reported only in isolated cases. The available evidence also suggests that disease severity is influenced by toxin absorption, the integrity of the castor bean seed coat, and the promptness of supportive treatment.

The studies included in this review [16, 17, 19] collectively illustrate these features while also emphasizing the diagnostic importance of ricinine as a biomarker of exposure. Gastrointestinal manifestations represent the hallmark of ricin ingestion and typically develop within several hours after exposure. Nausea, vomiting, abdominal pain, and diarrhea are the most frequently reported symptoms, although the severity of illness varies considerably depending on the amount of toxin absorbed.

A representative pediatric case was reported from Nepal [16], although only limited clinical details were available. More detailed information was provided by the adult case described by Steinacher et al. [19], involving a 23-year-old woman who intentionally ingested approximately 15 castor beans. Shortly after presentation, she developed a wide-complex tachycardia with alternating QRS morphology consistent with bidirectional ventricular tachycardia. This severe arrhythmia occurred in association with gastrointestinal symptoms, agitation, and probable aspiration, ultimately necessitating endotracheal intubation. This case is particularly noteworthy because it demonstrates that severe cardiac toxicity may occasionally dominate the clinical picture, expanding the recognized spectrum of ricin poisoning beyond gastrointestinal injury and systemic inflammatory manifestations.

Compared with injection exposure, outcomes following ingestion appear substantially more variable. While many patients recover with supportive treatment, fatal cases have also been documented. Conversely, the patient described by Steinacher et al. [19] survived despite experiencing life-threatening cardiac complications. Following intensive supportive management, including airway protection and hemodynamic stabilization, the arrhythmia resolved and the patient eventually recovered completely after a prolonged hospitalization. These findings suggest that aggressive supportive care may substantially improve outcomes even in severe ingestion-related poisonings.

The mixed-exposure case described by Røen et al. [17], involving both oral ingestion and injection, presents additional interpretative challenges. Although the patient died approximately 38 h after exposure, the relative contribution of the ingested dose cannot be accurately determined because systemic toxicity was likely dominated by the injected ricin. Consequently, conclusions regarding ingestion-specific toxicodynamics should be drawn cautiously from this case.

Supportive care remains the cornerstone of treatment for ricin poisoning. However, a specific anti-ricin antibody therapy (Ricimed^®^, Fabentech) has recently received marketing authorization in France for the emergency treatment of confirmed or strongly suspected acute ricin intoxication in combination with appropriate supportive care. Because all cases included in this review predate its clinical availability, none of the patients received this treatment. The Nepalese pediatric case [16] demonstrates that favorable outcomes may be achieved even in resource-limited settings through prompt supportive care alone. More detailed therapeutic information was provided in the case reported by Steinacher et al. [19]. Activated charcoal was administered in an attempt to reduce further gastrointestinal absorption, while intravenous sodium bicarbonate was used to correct metabolic acidosis and assist in arrhythmia management. Multiple electrical cardioversions and intravenous amiodarone failed to achieve immediate rhythm control. Nevertheless, the patient spontaneously converted to sinus rhythm after approximately two hours of continued supportive treatment, suggesting that toxin redistribution and physiological recovery may have contributed more substantially to clinical improvement than antiarrhythmic interventions alone. Recurrent episodes of non-sustained ventricular tachycardia and atrial fibrillation further support the hypothesis that ricin may exert direct cardiotoxic effects, a phenomenon that has been more frequently discussed in injection-related poisoning but remains relatively underreported following ingestion.

 Confirmation of ricin ingestion relies primarily on the detection of ricinine in biological specimens. It should be emphasized, however, that ricinine is a biomarker of exposure to castor beans rather than to purified ricin itself. Consequently, in scenarios involving highly purified ricin preparations, such as intentional criminal use or bioterrorism, ricinine may be absent or present only in negligible amounts. In such circumstances, laboratory confirmation requires the direct detection of ricin using specific analytical techniques, including immunological assays (e.g., ELISA), mass spectrometry–based methods, or functional assays capable of demonstrating the biological activity of the toxin. The mixed-exposure case described by Røen et al. [17] provided valuable quantitative information regarding ricinine kinetics. In that patient, serum ricinine concentrations decreased from approximately 33 ng/mL to 23 ng/mL between 10 and 29 h after exposure, whereas urinary concentrations ranged from 20 to 58 ng/mL. Peak urinary concentrations were observed between 12 and 29 h post-exposure, further supporting the role of renal elimination as the principal clearance pathway. However, available data on human ricinine pharmacokinetics remain limited and are derived from a small number of published case reports. Although these data must be interpreted cautiously because of the concomitant injection exposure, they provide a useful reference framework for the interpretation of ricinine concentrations in human poisoning cases.

The studies reviewed consistently highlight the importance of rapid ricinine detection as a diagnostic tool. This is particularly relevant in pediatric patients and in situations where a reliable exposure history is unavailable. Early identification of ricin poisoning may facilitate timely supportive management, prevent diagnostic delays, and reduce the need for unnecessary investigations aimed at more common infectious or toxicological conditions. These considerations are especially important in geographical regions where castor plants are widespread and accidental exposures remain possible. Consequently, the development of rapid analytical platforms, including point-of-care assays and highly sensitive LC–MS/MS methods for ricinine detection, should be regarded as a priority for both clinical toxicology and public health preparedness (Table 3 and 4).

**Table 3:** Summary of the ingestion exposure cases included in this review according to their clinical features, management strategies, and outcomes

Study	Patient Demographics	Presenting Symptoms	Cardiac Arrhythmia	Key Management	Outcome
[16]	Pediatric patient (Nepal)	Clinically affected, details not specified	Not reported	Basic supportive care	Not specified as fatal; diagnostic dilemma resolved
[17]	Adult (mixed injection and oral intake)	Not specified (died 38 h post-exposure)	Not reported	Intensive medical care (not specified)	Death
[19]	Previously healthy adult man	Severe agitation, suspected aspiration	Bidirectional ventricular tachycardia (180 bpm, alternating QRS axis); non-sustained VT and atrial fibrillation persisted after conversion	Activated charcoal, IV sodium bicarbonate, 5 electrical cardioversions, 450 mg IV amiodarone, endotracheal intubation, supportive care (~ 2 h)	Survival; intubated 10 days (aspiration pneumonia); discharged in good health at 24 days

**Table 4:** Biomarkers reported in human ricin poisoning and their potential diagnostic utility

Biomarker	Biological matrices	Diagnostic role	Strengths	Limitations
Ricinine	Blood, serum, urine, vitreous humour, bile, liver	Confirmation of ricin exposure	Most consistently detected biomarker; measurable by LC–MS/MS; useful ante- and postmortem	Limited human data for prognostic thresholds
Ricin A-chain	Serum	Direct evidence of toxin exposure	High specificity	Detected only in a limited number of reports; short detection window
IL-6, IL-10, TNF-α	Blood	Assessment of systemic inflammatory response	Reflect disease severity	Not specific for ricin poisoning
Troponin/CK	Blood	Assessment of myocardial injury	Useful for evaluating cardiac involvement	Non-specific biomarkers
Leukocytosis/thrombocytopenia	Blood	Supportive laboratory findings	Readily available	Non-specific

## Discussion

The evidence synthesized in this systematic review suggests a relatively consistent clinical characterization of fatal ricin poisoning. Despite differences in study design, sample size, reporting quality, and route of exposure, the available literature suggests the presence of a relatively consistent clinical pattern characterized by a progression from early nonspecific symptoms to shock, multiple organ failure, and death. Although the timing and severity of manifestations vary according to the route of exposure, injection-related poisonings consistently demonstrated a more rapid and severe clinical course than ingestion-related cases. Despite the limited number of available cases, several recurrent findings were observed that are noteworthy given the rarity of documented human ricin poisonings and the heterogeneous circumstances under which exposure occurred, including suicidal attempts, accidental ingestion, and homicidal attacks.

One of the most important findings emerging from this review is the consistent detection of ricinine in all studies that performed toxicological confirmation. Ricinine appeared to be a reliable biomarker of ricin exposure across multiple biological matrices, including serum, whole blood, urine, vitreous humour, bile, and liver tissue. Various analytical platforms, including LC–MS/MS, GC–MS, and HPLC, demonstrated excellent sensitivity and specificity, with reported detection limits as low as 50 pg/mL in whole blood [12]. The analytical performance of these methods, together with the relative stability of ricinine in biological specimens, supports its value as a preferred biomarker for confirming ricin exposure in both clinical and forensic settings.

Although ricinine emerged as the most consistently validated biomarker, other laboratory markers may provide complementary diagnostic information. Detection of ricin A-chain offers direct evidence of toxin exposure but has been reported only in a limited number of studies. Conversely, inflammatory cytokines (IL-6, IL-10, TNF-α) and markers of organ injury such as troponin or creatine kinase primarily reflect the host response and tissue damage rather than confirming exposure. Therefore, these biomarkers should be regarded as complementary rather than alternative tools, whereas ricinine currently remains the reference biomarker for laboratory confirmation of ricin poisoning.

The quantitative toxicological data reported by several studies also provide valuable insight into ricinine toxicokinetics. Serial measurements described by Røen et al. [17] and Verougstraete et al. [20] suggest a biphasic elimination pattern characterized by an initial rapid distribution phase followed by a slower elimination phase. Urinary ricinine concentrations were generally higher than corresponding serum concentrations, reflecting the predominant role of renal excretion in ricinine clearance. These findings are particularly relevant for forensic investigations, as they may assist in the interpretation of postmortem toxicological results and estimation of the interval between exposure and death. Although elevated ricinine concentrations appeared to be associated with more severe poisoning and rapid clinical deterioration in some cases [20], the limited number of available observations precludes the establishment of definitive prognostic thresholds.

These observations should nevertheless be interpreted cautiously. As highlighted by Lefever et al., current knowledge of ricinine pharmacokinetics is based on a very limited number of human cases, and no consistent correlation has been demonstrated between ricinine concentrations and poisoning severity. Therefore, ricinine should primarily be regarded as a biomarker of exposure rather than a validated prognostic biomarker [21].

Although ricinine remains the most practical biomarker in poisoning resulting from castor bean exposure, its diagnostic utility may be limited when purified ricin is used. In these situations, direct analytical detection of ricin becomes essential, particularly in forensic investigations involving suspected bioterrorism or deliberate criminal poisoning.

The clinical implications of these findings are substantial. Ricin poisoning should be considered in the differential diagnosis of patients presenting with acute gastrointestinal symptoms, rapidly progressive shock, and multiple organ dysfunction, particularly when conventional diagnostic investigations fail to identify an infectious or toxicological cause. The studies reviewed demonstrate that ricin poisoning can mimic several common medical conditions, most notably septic shock. This overlap is further complicated by the inflammatory response induced by ricin, which may include fever, leukocytosis, thrombocytopenia, and elevated circulating cytokine concentrations. Consequently, clinicians may initially attribute the presentation to severe bacterial infection, potentially delaying the correct diagnosis. The cases reported illustrates the diverse clinical manifestations associated with ricin toxicity, demonstrating that severe cardiac arrhythmias, including bidirectional ventricular tachycardia, may occasionally represent a dominant feature of poisoning [19]. These observations reinforce the importance of maintaining a high index of suspicion and obtaining appropriate biological specimens for toxicological analysis whenever ricin exposure is suspected.

From a therapeutic perspective, supportive care remains the cornerstone of management. Recently, a specific anti-ricin antibody therapy (Ricimed^®^, Fabentech) received marketing authorization in France for the treatment of confirmed or strongly suspected acute ricin poisoning. However, because all the cases included in this review occurred before its availability, supportive care remained the only therapeutic option described in the published literature. Current treatment is largely supportive and focuses on airway protection, fluid resuscitation, hemodynamic stabilization, correction of metabolic disturbances, and management of organ dysfunction. Nevertheless, the survival of several severely poisoned patients demonstrates that recovery remains possible when supportive measures are instituted promptly and aggressively. Early recognition therefore remains the most important determinant of clinical outcome.

The findings of this review also have important implications for forensic pathology. Although detailed autopsy descriptions were available only in a limited number of studies, a relatively consistent pattern of pathological findings emerged. Frequently reported observations included hemorrhagic gastrointestinal injury, hepatic necrosis, renal tubular damage, pulmonary edema, and serosal effusions, collectively supporting the hypothesis that endothelial injury and capillary leak syndrome play a central role in the pathophysiology of fatal ricin poisoning. The detection of ricinine in postmortem tissues, including vitreous humour, liver tissue, and bile, further strengthens its value as a forensic biomarker, particularly in cases where conventional specimens such as blood or urine are unavailable. Moreover, the relatively homogeneous postmortem distribution of ricinine described in some studies suggests that multiple tissues may be suitable for toxicological confirmation. However, the limited number of detailed autopsy investigations currently available highlights the need for standardized postmortem sampling protocols that include blood, urine, vitreous humour, bile, liver tissue, and, when appropriate, tissue from the suspected injection site.

Ricin poisoning also presents significant medico-legal challenges. The nonspecific clinical presentation and its resemblance to septic shock or other toxic syndromes may complicate both clinical diagnosis and forensic investigation. In the absence of a known exposure history, cases may remain unrecognized unless targeted toxicological testing is performed. This issue is particularly important given the historical use of ricin in homicidal attacks and its continued relevance as a potential bioterrorism agent. The cases identified in this review demonstrate the diverse circumstances under which ricin poisoning may occur, including accidental exposures, suicidal attempts, and deliberate criminal acts. Consequently, the confirmation of ricin exposure should prompt not only appropriate medical management but also a thorough forensic and public health investigation aimed at identifying the source of exposure and preventing additional cases.

Several limitations should be considered when interpreting the findings of this review. First, the available evidence remains limited and is derived predominantly from case reports and small case series, restricting the generalizability of the conclusions and precluding quantitative meta-analysis. Second, publication bias is likely, as unusual or severe cases are more likely to be reported than mild or diagnostically uncertain presentations. Third, restricting the review to English-language publications may have resulted in language bias and the exclusion of relevant reports from regions where castor plants are endemic. Fourth, substantial heterogeneity in reporting quality, clinical detail, toxicological analysis, and pathological investigation limited the availability of comparable data across studies. Finally, the absence of documented human inhalational poisoning cases represents a major gap in the literature, particularly given the longstanding concern regarding the potential aerosolization of ricin in bioterrorism scenarios.

An additional source of uncertainty derives from the heterogeneity of the available evidence. The included studies differed substantially with respect to exposure route, ricin preparation, administered dose, timing of clinical presentation, analytical techniques used for toxicological confirmation, and the completeness of clinical and pathological reporting. These differences limited direct comparisons between studies and precluded quantitative synthesis or formal meta-analysis. Consequently, the conclusions of this review should be interpreted as a qualitative synthesis of the currently available.

Also, because this review deliberately focused on fatal and near-fatal human poisonings to address specific medico-legal and forensic questions, its findings should not be interpreted as representative of the entire clinical spectrum of ricin intoxication.

The review also identifies several priorities for future research. Large multicenter studies incorporating standardized clinical, toxicological, and pathological data collection are urgently needed to strengthen the evidence base. Such investigations could facilitate the establishment of reference and potentially prognostic ricinine concentrations across different biological matrices. Additional research should focus on the development of rapid diagnostic assays suitable for emergency and point-of-care settings, as well as on the validation of alternative sampling approaches such as dried blood spots and dried urine spots. Standardized postmortem toxicology protocols should also be developed to improve inter-study comparability and facilitate forensic interpretation. Furthermore, a better understanding of human ricin toxicokinetics following different exposure routes, particularly inhalation, remains an important unmet research need. Finally, continued efforts toward improving and validating targeted therapeutic interventions, including next-generation monoclonal antibodies and other anti-ricin strategies, remain warranted.

## Conclusions

This systematic review integrates the currently available evidence on fatal ricin poisoning and suggests the presence of a relatively consistent clinicopathological pattern characterized by rapid progression from early gastrointestinal and systemic symptoms to shock, multiple organ failure, and death. Although this progression may occur following different routes of exposure, injection-related poisonings consistently exhibited a more rapid and severe course than ingestion-related cases.

Across the studies included in this review, ricinine appears to be the most consistently validated biomarker of exposure currently available. Its detection in multiple biological matrices provides robust confirmation of ricin exposure, although further studies are needed to better define its relationship with exposure timing and clinical severity. These findings suggest an important role of ricinine analysis in both clinical toxicology and forensic pathology.

Clinicians should maintain awareness of ricin poisoning in patients presenting with unexplained gastrointestinal illness, rapidly progressive shock, and multiple organ dysfunction, particularly when infectious causes cannot fully explain the clinical picture. Early recognition and prompt supportive treatment remain essential. Although a specific anti-ricin antibody therapy has recently become available in France, none of the cases included in this review received this treatment because they predated its clinical introduction. Forensic pathologists may benefit from the incorporation of ricinine testing into postmortem investigations of unexplained deaths associated with shock, organ failure, or suspicious circumstances.

The inclusion of studies published up to June 2026 confirms that ricin poisoning continues to attract scientific and forensic attention, particularly in relation to advances in analytical toxicology, biomarker identification, and postmortem investigation. Nevertheless, important knowledge gaps remain, including the lack of standardized postmortem sampling protocols, limited information regarding lethal ricinine concentrations, and the scarcity of human data on inhalational exposure. Addressing these gaps through collaborative multicenter research will be essential for improving diagnosis, forensic investigation, clinical management, and public health preparedness in future cases of ricin poisoning.

## Supplementary Information

Below is the link to the electronic supplementary material.

Supplementary Material 1 (DOCX 13.2 KB)

Supplementary Material 2 (DOCX 13.8 KB)

## Data Availability

No datasets were generated or analysed during the current study.
