# Supplementary material for: Medico-legal aspects of fatal ricin poisoning: a systematic review of clinical manifestations, toxicological findings and autopsy results
Source: Int J Legal Med. 2026 Aug 6;140(6):3597–609. doi: 10.1007/s00414-026-03949-0 (PMC13658434; doi:10.1007/s00414-026-03949-0)
Supplement: Supplementary file 1 [file 414_2026_3949_MOESM1_ESM.docx]

**Supplementary Table S1. Representative database search strategies.**

| **Database** | **Representative search strategy** |
| --- | --- |
| **PubMed** | ((Ricin OR "Ricinus communis" OR "castor bean") AND (poisoning OR intoxication OR toxicity OR fatal OR death) AND (clinical manifestations OR symptoms OR toxicokinetics OR biomarkers) AND (autopsy OR pathology OR postmortem OR histopathology) AND (forensic OR medico-legal OR legal implications OR criminal)) |
| **Scopus** | TITLE-ABS-KEY (((Ricin OR "Ricinus communis" OR "castor bean") AND (poisoning OR intoxication OR toxicity OR fatal OR death) AND (clinical manifestations OR symptoms OR toxicokinetics OR biomarkers) AND (autopsy OR pathology OR postmortem OR histopathology) AND (forensic OR medico-legal OR legal implications OR criminal))) |
| **Web of Science** | TS=(((Ricin OR "Ricinus communis" OR "castor bean") AND (poisoning OR intoxication OR toxicity OR fatal OR death) AND (clinical manifestations OR symptoms OR toxicokinetics OR biomarkers) AND (autopsy OR pathology OR postmortem OR histopathology) AND (forensic OR medico-legal OR legal implications OR criminal))) |
