# Supplementary material for: Medico-legal aspects of fatal ricin poisoning: a systematic review of clinical manifestations, toxicological findings and autopsy results
Source: Int J Legal Med. 2026 Aug 6;140(6):3597–609. doi: 10.1007/s00414-026-03949-0 (PMC13658434; doi:10.1007/s00414-026-03949-0)
Supplement: Supplementary file 2 [file 414_2026_3949_MOESM2_ESM.docx]

**Supplementary Table S2. Methodological quality assessment of the included studies according to the modified Joanna Briggs Institute (JBI) Critical Appraisal Checklist.**

| **Study** | **Study design** | **Overall quality** | **Risk of bias** | **Main limitations** |
| --- | --- | --- | --- | --- |
| Aviram et al. (2022) | Case report | High | Low | Limited to a single case |
| Bucaretchi et al. (2020) | Case report | High | Low | Single patient |
| Coopman et al. (2009) | Case report | Moderate | Moderate | Limited toxicological and pathological information |
| Passeron et al. (2004) | Case report | Moderate | Moderate | Incomplete clinical follow-up |
| Rauniyar et al. (2025) | Case report | Moderate | Moderate | Limited clinical and laboratory details |
| Røen et al. (2013) | Case report | High | Low | Mixed exposure route |
| Staňková et al. (2020) | Case report | High | Low | Single case |
| Steinacher et al. (2026) | Case report | High | Low | Single patient |
| Verougstraete et al. (2019) | Case report | High | Low | Single case |
